# An Efficient Method of Birch Ethanol Lignin Sulfation with a Sulfaic Acid-Urea Mixture

**DOI:** 10.3390/molecules27196356

**Published:** 2022-09-26

**Authors:** Alexander V. Levdansky, Natalya Yu. Vasilyeva, Yuriy N. Malyar, Alexander A. Kondrasenko, Olga Yu. Fetisova, Aleksandr S. Kazachenko, Vladimir A. Levdansky, Boris N. Kuznetsov

**Affiliations:** 1 Institute of Chemistry and Chemical Technology, Krasnoyarsk Science Center, Siberian Branch, Russian Academy of Sciences, Akademgorodok 50/24, 660036 Krasnoyarsk, Russia; 2 School of Non-Ferrous Metals and Material Science, Siberian Federal University, Pr. Svobodny 79, 660041 Krasnoyarsk, Russia

**Keywords:** birch ethanol lignin, sulfamic acid, urea, sulfation process optimization, sulfated product characterization, FTIR spectroscopy, 2D NMR spectroscopy, gel permeation chromatography, thermal analysis

## Abstract

For the first time, the process of birch ethanol lignin sulfation with a sulfamic acid-urea mixture in a 1,4-dioxane medium was optimized experimentally and numerically. The high yield of the sulfated ethanol lignin (more than 96%) and containing 7.1 and 7.9 wt % of sulfur was produced at process temperatures of 80 and 90 °C for 3 h. The sample with the highest sulfur content (8.1 wt %) was obtained at a temperature of 100 °C for 2 h. The structure and molecular weight distribution of the sulfated birch ethanol lignin was established by FTIR, 2D ^1^H and ^13^C NMR spectroscopy, and gel permeation chromatography. The introduction of sulfate groups into the lignin structure was confirmed by FTIR by the appearance of absorption bands characteristic of the vibrations of sulfate group bonds. According to 2D NMR spectroscopy data, both the alcohol and phenolic hydroxyl groups of the ethanol lignin were subjected to sulfation. The sulfated birch ethanol lignin with a weight average molecular weight of 7.6 kDa and a polydispersity index of 1.81 was obtained under the optimum process conditions. Differences in the structure of the phenylpropane units of birch ethanol lignin (syringyl-type predominates) and abies ethanol lignin (guaiacyl-type predominates) was manifested in the fact that the sulfation of the former proceeds more completely at moderate temperatures than the latter. In contrast to sulfated abies ethanol lignin, the sulfated birch ethanol lignin had a bimodal and wider molecular weight distribution, as well as less thermal stability. The introduction of sulfate groups into ethanol lignin reduced its thermal stability.

## 1. Introduction

Lignin is the most widespread aromatic polymer on the earth. The lignin content in the dry mass of woody plants ranges within 15–40%, depending on the species [1,2]: 5–12% in herbaceous plants, 25–35% in coniferous wood, and 15–30% in deciduous wood. Lignin has an irregular 3D structure built from phenylpropane units (PPUs) with different numbers of methoxyl groups in the aromatic ring: p-hydroxyphenyl (H), guaiacyl (G), and syringyl (S). The PPUs are randomly cross-linked with simple ether C–O–C and C–C bonds [3]. In addition, the propane chains of the lignin PPUs can contain different functional groups: hydroxyl (–OH), carbonyl (C=O), carboxyl (–COOH), and double bonds (–CH=CH–).

The structure, chemical composition, and physicochemical properties of lignins vary within fairly wide limits, depending on the lignocellulosic raw material and lignin isolation method used [4]. The complex heterogeneous composition of lignins complicates the development of efficient techniques for utilization to obtain valuable products [5].

Recently, there has been an increased interest in organosolv methods for extracting cellulose from lignocellulosic biomass. The use of organic solvents makes the organosolv processes environmentally friendly and eliminates the contamination of lignin with sulfur [6]. Organosolv lignins have a higher content of hydroxyl and carbonyl groups [7] than conventional technical lignins and are soluble in organic solvents, which facilitates the chemical modification and processing of these lignins [8,9,10].

A promising direction in the valorization of lignin is its chemical modification to obtain bioactive derivatives [11,12,13]. In particular, sulfated lignins are known for their anticoagulant and antiplatelet activity and can be used in the treatment of thrombotic disorders [11].

The available methods for obtaining the sulfated lignin derivatives are based on the use of aggressive and environmentally hazardous sulfating reagents, e.g., sulfuric anhydride and its complexes with toxic amines [13,14]. The method for the enzymatic sulfation of organosolv and technical lignins was proposed in [15] and is based on the use of p-nitrophenyl sulfate (p-NPS) as a sulfate donor and aryl sulfotransferase (AST) as a catalyst. This method showed high selectivity for the phenolic hydroxyl groups, leaving the aliphatic hydroxyl groups in the lignin side chain intact. The main drawback of this method is the long sulfation time (96 h). We developed a new, simpler, environmentally safe method for producing water-soluble abies ethanol lignin sulfates, which uses low-toxic sulfamic acid mixed with urea as a sulfating agent [16]. The comparative 2D NMR spectroscopy analysis of the structure of the initial and sulfated abies ethanol lignins was used to establish the main structural units and moieties of lignin macromolecules.

It is known well that, in contrast to lignins of abies and other coniferous species, which consist mainly of the guaiacyl structural units, in the hardwood (e.g., birch) lignin structure, the syringyl units dominate [17,18]. Coniferous and hardwood lignins also contain different amounts of condensed structural units; hardwood lignins are less condensed [4]. Birch is widespread in Russia and other countries of the Northern Hemisphere, but its wood finds only limited application in the pulp, paper, and building industries. However, the high content of xylan in birch wood allows it to carry out the complex processing of its biomass with the production of xylose, levulinic acid, and ethanol lignin [19]. Birch ethanol lignin contains no sulfur and has a relatively low molecular weight and a fairly narrow molecular weight distribution, which makes it a convenient substrate for the synthesis of bioactive lignin sulfates.

The aim of this study was to experimentally and numerically optimize the process of the sulfation of ethanol lignin birch wood with a mixture of sulfamic acid and urea in a 1,4-dioxane medium and to characterize the structure and thermochemical properties of the sulfated ethanol lignin.

## 2. Results

### 2.1. Kinetic Study of the Process of Birch Ethanol Lignin Sulfation

**Figure 1:**
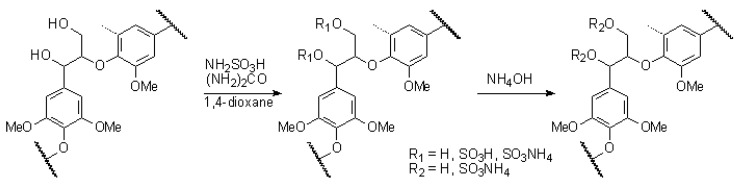
Scheme of sulfation of ethanol lignin with the sulfamic acid–urea mixture in 1,4-dioxane medium using β-aryl ethers (β-O-4′) lignin moieties as an example.

The scheme of birch ethanol lignin sulfation with the low-toxic sulfamic acid–urea mixture in the 1,4-dioxane medium is shown in Figure 1. The isolation of sulfated lignin was isolated in the form of an ammonium salt.

The use of the low-toxic and non-corrosive mixture of sulfamic acid and urea for the sulfation of abies ethanol lignin was previously proposed [16]. The yield of sulfated ethanol lignin of abies wood and sulfur content can be regulated by varying the sulfation process temperature, time, and the ratio of lignin to sulfating complex (sulfamic acid and urea mixture).

A high yield of sulfated lignin and sulfur content can be obtained at different combinations of the above-mentioned parameters of the sulfation process. Taking into account the data obtained when optimizing the process of abies ethanol lignin sulfation with a mixture of sulfamic acid and urea, the ratio of birch ethanol lignin to the sulfating complex was chosen in this study to be 1:3 mol/mol.

The sulfation process temperature ranged from 70 to 110 °C and the process time from 0.5 to 3.0 h. The data on the effect of the birch ethanol lignin sulfation conditions on the yield of sulfated lignin and sulfur content are given in Table 1.

The high yield of sulfated birch ethanol lignin and the high sulfur content can be obtained at different combinations of the specified parameters of the sulfation process (temperature and time).

The sulfated ethanol lignin samples with a high yield (91.1–96.4%) and a sulfur content of 7.1–8.1 wt % were obtained at a process temperature of 90 °C and a time of 3 h or at a temperature of 100 °C and a time of 1.5–2.0 h. A further increase in the sulfation time and temperature did not significantly affect the sulfur content in the product but reduced the sulfated lignin yield. This may be due to the intensification of secondary condensation and destruction reactions under more severe conditions, which leads to the formation of products that are removed at the stage of sulfated lignin dialysis. It should be noted that the products of sulfation of birch ethanol lignin contain somewhat more sulfur than the products of sulfation of abies ethanol lignin under similar conditions [16]. This is possibly related to the lower reactivity of coniferous lignins that contain more condensed structural units than hardwood lignins [4].

**Figure 2:**
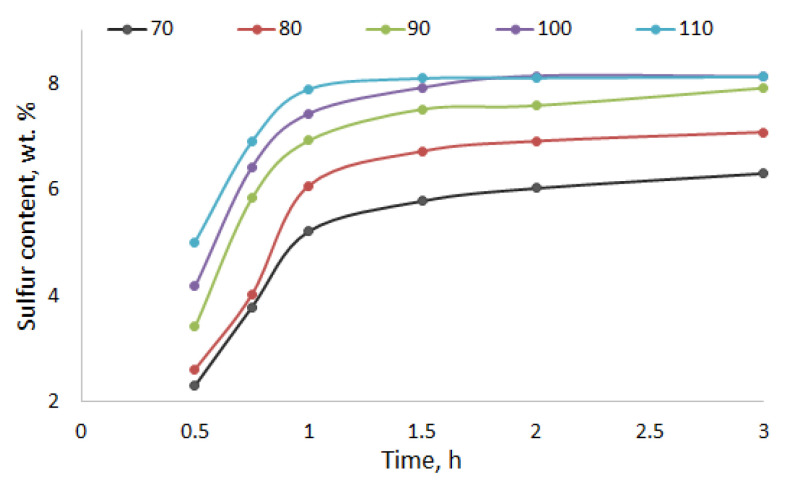
Dynamics of the sulfur content in the process of birch ethanol lignin sulfation with sulfamic acid–urea mixture at different temperatures.

**Figure 3:**
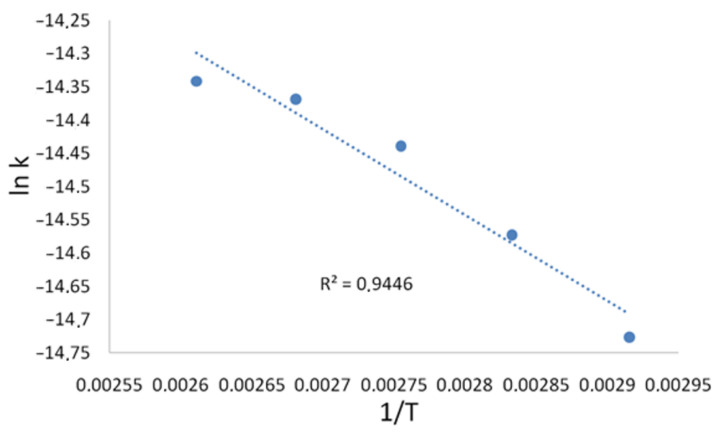
Temperature dependence of the rate constants of the birch ethanol lignin sulfation process.

The kinetics of the birch ethanol lignin sulfation with sulfamic acid–urea mixture in 1,4-dioxane medium was investigated in the temperature range of 70–100 °C (Figure 2). The apparent rates of the birch ethanol lignin sulfation were calculated from the change in the sulfur content in sulfated ethanol lignin. The calculation was made using the first-order equation. The activation energy of the sulfation process was determined from the temperature dependence of the rate constants in the Arrhenius coordinates (Figure 3).

The calculated apparent rate constants and activation energies of the birch ethanol lignin sulfation process are given in Table 2.

The activation energies of the processes of sulfation of birch and abies ethanol lignins with sulfamic acid–urea mixture in 1,4-dioxane medium are similar: 10.7 kJ/mol for birch lignin (Table 2) and 8.4 kJ/mol for abies lignin [16]. It should be noted that, for the process of starch sulfation in a deep eutectic solvent (the sulfamic acid–urea mixture), the activation energy was 6.4 kJ/mol [20] and, for the sulfation of arabinogalactan with sulfamic acid in DMSO it was 13.1 kJ/mol [21]. It is known that the low activation energy of the process may indicate the presence of significant diffusion restrictions [22,23]. Taking this into account, we can conclude that, under the chosen conditions, the processes of biopolymer sulfation proceed under diffusion restrictions.

The solubility of the sulfated lignin in water increased with an increase in the content of sulfate groups. The maximum sulfur content in the sulfated ethanol lignin was estimated to be 10.6 wt %, taking into account the hypothetical structure of the Berkman spruce lignin [4], in which one phenylpropane unit has 0.9 mol of free OH groups capable of sulfating. In order to find the conditions that ensure the production of sulfated birch ethanol lignin with maximum yield and sulfur content, a numerical optimization of the sulfation process was carried out.

### 2.2. Numerical Optimization of the Process of Birch Ethanol Lignin Sulfation

As independent variables, we used two factors: process temperature *X*_1_ (70, 80, 90, 100, 110 °C) and time *X*_2_ (0.5, 0.75, 1.0, 1.5, 2.0, 3.0 h). The result of the sulfation process was characterized by two output parameters: sulfur content *Y*_1_ (wt %) in the sulfated ethanol lignin and sulfated ethanol lignin yield *Y*_2_ (wt %). The fixed parameter was the ratio L:SC = 1:3. A combined multilevel experiment plan (Users Design) was used in the calculations. The designations of the variables are listed in Table 3.

The experimental results given in Table 1 were used in the mathematical processing and optimization of the birch ethanol lignin sulfation process.

The dependences of the output parameters on the variable process factors were approximated by second-order regression equations. The results of the variance analysis are given in Table 4.

The variance analysis showed that, within the limits of the experimental conditions used, the greatest contribution to the total variance of the output parameter was made by both factors: the temperature and time of the birch ethanol lignin sulfation process. This is indicated by the high variance ratios (*F*) for the main effects, which are called also the influence efficiencies. The data in the columns of Table 4 (*P*) are interpreted similarly. The influence of the variance source on the output parameter is considered to be statistically significant if its significance level is lower than a specified critical value (in our case, 0.05).

The dependence of the sulfur content *Y*_1_ in the sulfated birch ethanol lignin on the process variables is approximated by the regression equation

(1) $$\begin{array}{l} Y_{1}=-12.7119+0.3212\times X_{1}+3.27557\times X_{2}-0.00149576\times X_{1}{}^{2}+\\ +0.000225435\times X_{1}\times X_{2}-0.693718\times X_{2}{}^{2} \end{array}$$

**Figure 4:**
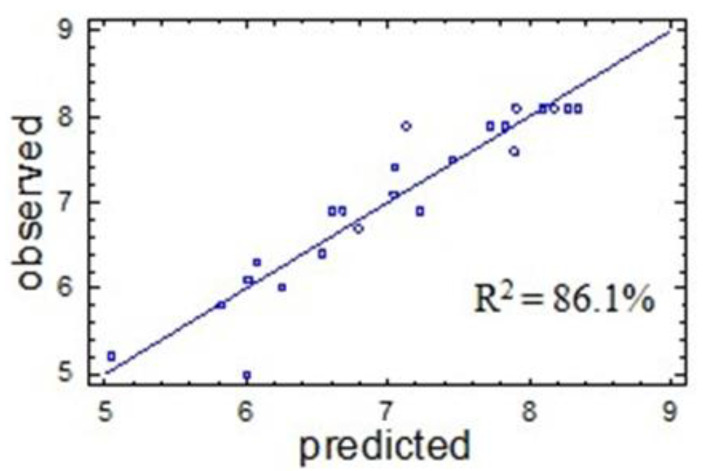
Output parameter *Y*_1_ observed in the experiment (dots) and predicted by mathematical model (1) (solid line).

The predictive properties of Equation (1) are illustrated in Figure 4, in which the experimental values of the output parameter *Y*_1_ are compared with its values calculated using Equation (1). The straight line corresponds to the calculated *Y*_1_ values, and the dots correspond to the observed values. The proximity of the experimental points to the straight line confirms the good predictive properties of Equation (1).

The approximation quality is characterized also by the determination coefficient *R*^2^_*adj*_. In the case under consideration, the value is *R*^2^_*adj*_ = 86.1%, which indicates acceptable approximation quality. This confirms the adequacy of Equation (1) for the experiment and makes it possible to use this equation as a mathematical model of the process under study.

**Figure 5:**
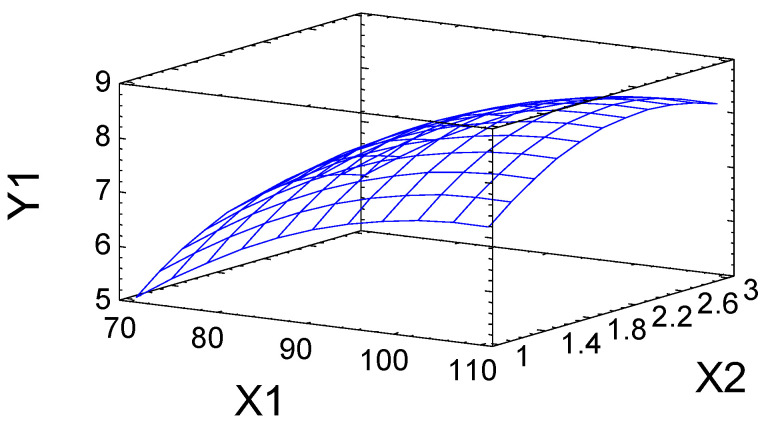
Response surface illustrating the dependence of sulfur content (wt %) in sulfated birch ethanol lignin on the process temperature (*X*_1_) and time (*X*_2_).

Using the mathematical model, the dependence of the output parameter *Y*_1_ on the variables *X*_1_ and *X*_2_ was plotted in the form of a response surface (Figure 5).

According to the calculation using mathematical model (1), the maximum predicted sulfur content (8.4 wt %) was obtained at the point corresponding to a process temperature of 107 °C and a process time of 2.3 h.

According to the results of the variance analysis within the limits of the chosen experimental conditions, the sulfation temperature contributes significantly to the total variance of the output parameter *Y*_2_ (sulfated lignin yield, wt %). This is indicated by the high variance relation (*F*) corresponding to this factor and the small *P* criterion.

The dependence of *Y*_2_ on the variable process factors is approximated by the regression equation

(2) $$\begin{array}{l} Y_{2}=36.8673+1.29494\times X_{1}+7.57733\times X_{2}-0.00715242\times X_{1}{}^{2}-\\ -0.0850839\times X_{1}\times X_{2}-0.14729\times X_{2}{}^{2} \end{array}$$

**Figure 6:**
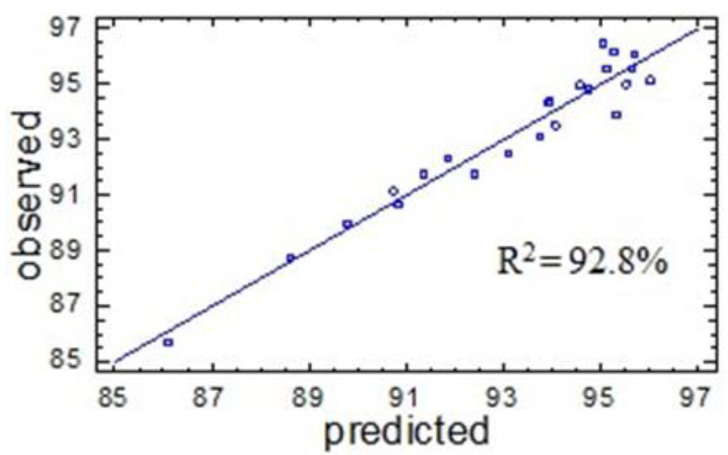
Comparison of the values of output parameter *Y*_2_ observed in the experiment and those predicted by Equation (2).

The determination coefficient is fairly high, *R*^2^_*adj*_ = 92.8%, which evidences the good approximation quality. The latter is also confirmed by the good agreement between the output parameters calculated using Equation (2) and those obtained in the experimental measurements. This confirms the adequacy of Equation (2) for the experiment and its use as a mathematical model of the process under study (Figure 6).

**Figure 7:**
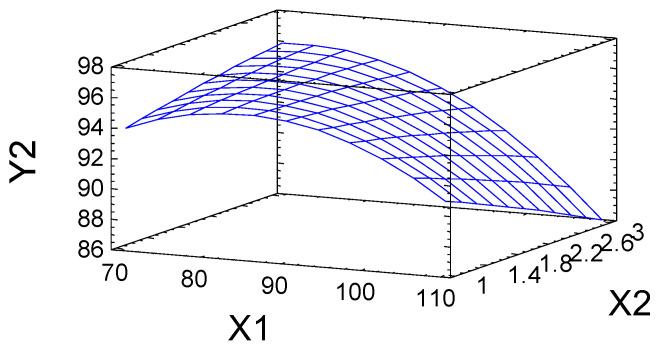
Response surface of the dependence of the sulfated birch ethanol lignin yield on the variable temperature (*X*_1_) and time factors (*X*_2_).

Figure 7 shows the graphical representation of the dependence of the sulfated birch ethanol lignin yield on the variable factors *X*_1_ and *X*_2_.

According to the above-described model, the optimum conditions for the sulfation of birch ethanol lignin that ensure the maximum yield of sulfated birch ethanol lignin (96.1 wt %) correspond to a process temperature of 78 °C and a time of 2.9 h.

### 2.3. Characterization of the Sulfated Birch Ethanol Lignin

The substitution of hydroxyl groups for sulfate groups during the sulfation of the birch ethanol lignin with the sulfamic acid–urea mixture was confirmed by FTIR and NMR spectroscopy.

**Figure 8:**
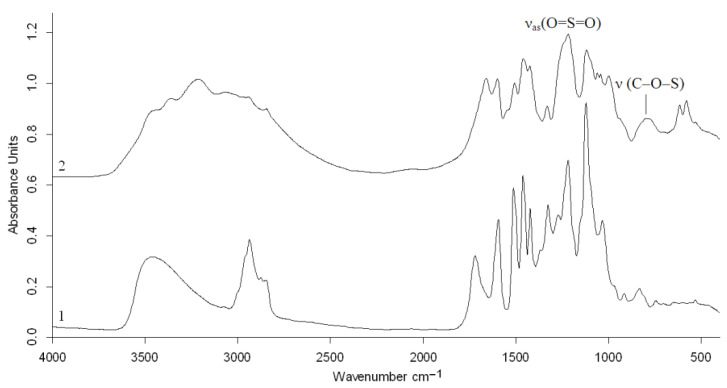
FTIR spectra of birch ethanol lignin (1) and sulfated ethanol lignin ammonium salt (2).

The FTIR spectrum of birch ethanol lignin (Figure 8) contains absorption bands characteristic of hardwood lignins (GS) [24]. The band at 1123 cm^−1^ corresponds to planar bending vibrations of the C–H syringyl aromatic rings, and the C–O stretching vibrations in secondary alcohols are dominant in the spectrum. The pronounced band with a maximum at 1327 cm^−1^ belongs to the skeletal vibrations of the syringyl ring with the C–O stretching vibrations. In addition, the spectrum includes medium-intensity absorption bands around 1271 and 1034 cm^−1^, characteristic of the vibrations of the guaiacyl units of lignin [4].

In contrast to the spectrum of the initial ethanol lignin, the FTIR spectrum of the ammonium salts of the ethanol lignin sulfates contained a new absorption band at 798 cm^−1^ (Figure 8), which corresponds to the stretching vibrations of the C–O–S bond of the sulfate group and a broad absorption band with the maximum at 1218 cm^−1^ corresponding to the asymmetric stretching vibrations υ_as_(O=S=O).

The FTIR spectra of birch and abies ethanol lignin sulfates [16] obtained in a similar way are almost identical, except for the presence in the spectrum of the sulfated birch ethanol lignin of the adsorption band at 1331 cm^−1^ characteristic of the syringyl structures.

**Figure 9:**
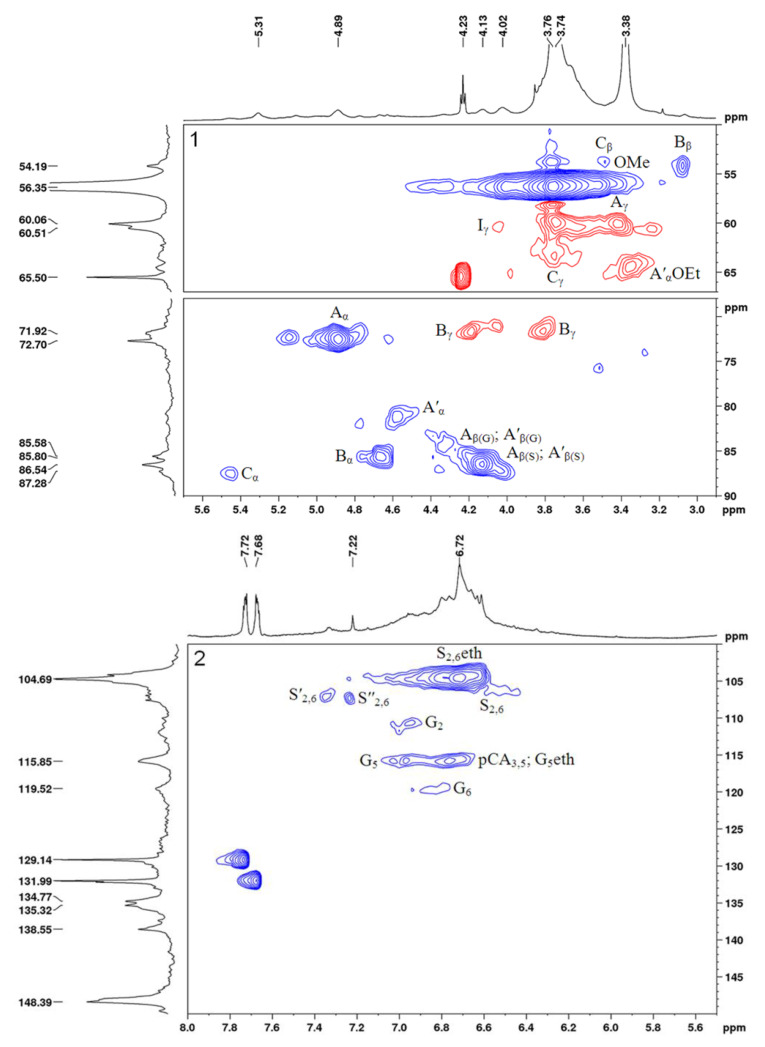
HSQCed spectrum of ethanol lignin: aliphatic oxygenated region 1 and aromatic region 2. The assignment of signals is given in Table 5, and the main identified structural units and fragments are shown in Figure 11.

**Figure 10:**
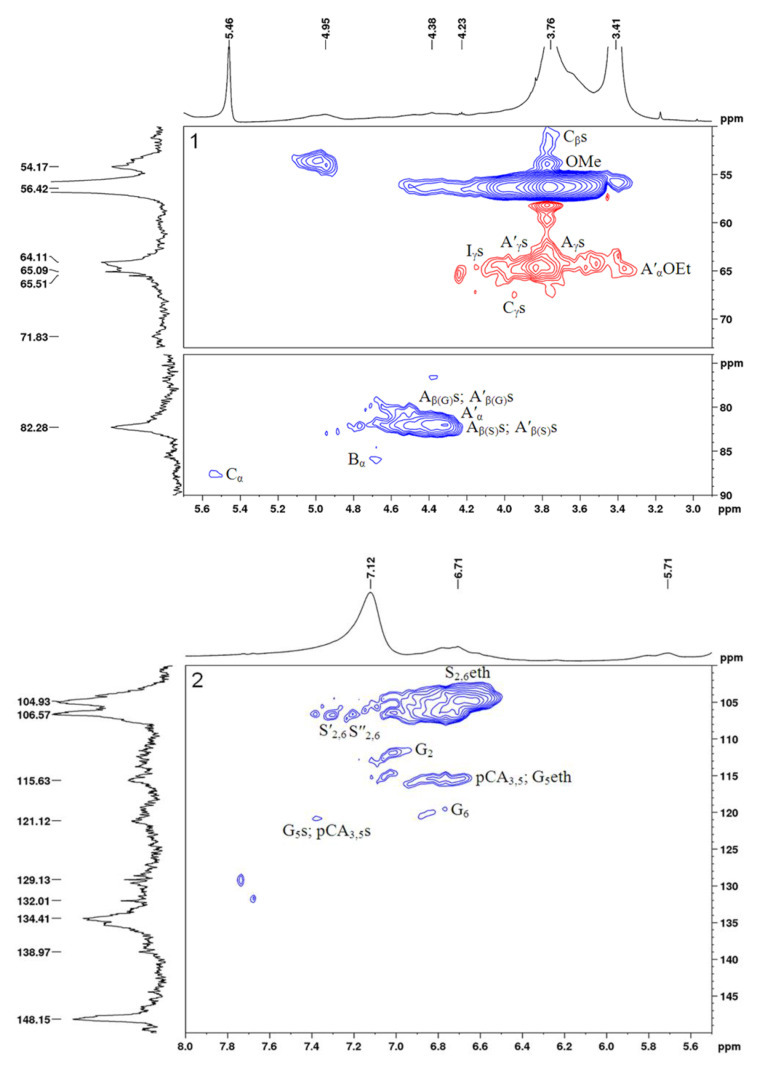
HSQCed spectrum of sulfated ethanol lignin: aliphatic oxygenated region 1 and aromatic region 2. The assignment of signals is given in Table 5, and the main identified structural units and fragments are shown in Figure 11.

**Figure 11:**
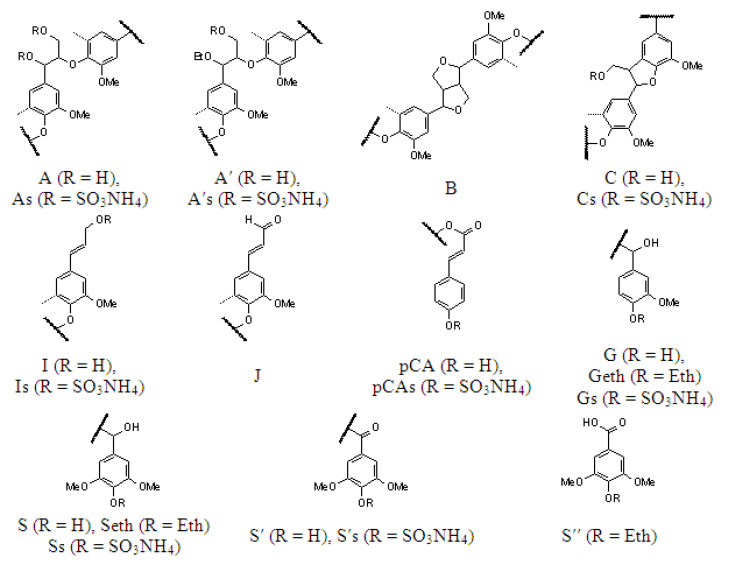
Main structural units and moieties of the initial and sulfated ethanol lignins: (**A**) β-aryl ethers, (**As**) α,γ-sulfated (α,γ-COSO_3_NH_4_) β-aryl ethers, (**A′**) α-ethoxylated (α-COEt) β-aryl ethers, (**A′s**) γ-sulfated (γ-COSO_3_NH_4_) α-ethoxylated (α-COEt) β-aryl ethers, (**B**) pinoresinols, (**C**) phenylcoumarans, (**Cs**) γ-sulfated (γ-COSO_3_NH_4_) phenylcoumarans, (**I**) cinnamyl alcohol end groups, (**Is**) γ-sulfated (γ-COSO_3_NH_4_) cinnamyl alcohol end groups, (**J**) cinnamyl aldehyde end groups, (**pCA**) p-coumarates, (**pCAs**) 4-sulfated (4-COSO_3_NH_4_) p-coumarates, (**S**) syringyl units, (**Seth**) 4-etherified (4-COEth) syringyl units, (**S′**) oxidized (α-C=O) syringyl units, (**S″**) oxidized (α-COOH) syringyl units, (**G**) guaiacyl units, (**Geth**) 4-etherified (4-COEth) guaiacyl units, and (**Gs**) 4-sulfated (4-COSO_3_NH_4_) guaiacyl units.

The 2D HSQC NMR spectra of the initial and sulfated birch ethanol lignins are shown in Figure 9 and Figure 10, respectively. The main ^1^H–^13^C peaks in the HSQC spectra identified using the literature data [25,26,27] are given in Table 5, together with the chemical shifts of some low-intensity peaks (not shown in Figure 9 and Figure 10). The main structural units and fragments of the initial and sulfated birch ethanol lignins are presented in Figure 11.

The HSQC spectra of the initial and sulfated ethanol lignin samples were compared in the regions of the chemical shifts of atoms from the lignin side chains (δ_C_/δ_H_ 50–90/2.9–5.7 ppm) and aromatic rings (δ_C_/δ_H_ 100–150/5.5–8.0 ppm).

Considering the region of the ^1^H–^13^C side chain signals in the HSQC spectrum of the birch ethanol lignin (Figure 9), we can see that it contains the intense correlation peaks of β-aryl ethers (A), pinoresinol (B), and phenylcoumaran fragments (C) (see Figure 11). A part of β-aryl ethers is ethoxylated in the α-position, judging by the fact that the spectra contain signals of the methylene group in the α-ethoxylated β-O-4′ bonds (δ_C_/δ_H_ 64.4/3.33) and the α-position of the α-acylated β-O-4′ bonds (δ_C_/δ_H_ 81.2/4.56). This assumption is confirmed by the presence of a correlation signal of the methyl group at δ_C_/δ_H_ 14.3/1.00 ppm.

In the spectrum of the sulfated ethanol lignin (Figure 10), the group of peaks assigned to the phenylcoumaran fragments (C) is characterized by a change in the position of the correlation signals Cγ-H_γ_ and Cβ-H_β_ (Figure 10), which is related to the effect of sulfation of OH groups in the γ position (Cγ-H_γ_: a shift of Δδ_C_ ~ 4 ppm toward weak fields; Cβ-H_β_: a shift of Δδ_C_ ~ 3 ppm toward strong fields).

A similar change in the peak positions along the carbon atom axis in the sulfated ethanol lignin spectrum is observed for β-aryl ethers β-O-4′ (A) and α-ethoxylated β-aryl ethers (A′). The Cγ-H_γ_ correlation signals of the sulfated lignin are shifted relative to the initial lignin signals toward weak fields by ~5 ppm and the Cβ-H_β_ signals toward strong fields by ~4 ppm. Such shifts of the signals in the spectra are most likely due to the sulfation of OH groups of β-aryl ethers of the lignin macromolecule in the γ position. In addition, free OH groups in the α position of β-aryl ethers (A) are probably subjected to the sulfation, since the Cα-H_α_ peak at δ_C_/δ_H_ 72.5/4.88 ppm is missing in the sulfated lignin spectrum.

The shift of signals in the aliphatic region of the spectrum of the sulfated lignin sample as compared with the spectrum of the initial lignin was also found for the C_γ_–H_γ_ correlations of the cinnamyl alcohol end groups (I). This is also indicative of the sulfation of OH groups bonded with carbon atoms C_γ_ of this lignin fragment.

Despite the presence of the fairly intense Cα-H_α_, Cβ-H_β_, and Cγ-H_γ_ signals of pinoresinol (β–β′) fragments (B) in the spectrum of the initial ethanol lignin, the intensity of these signals in the spectrum of the sulfated sample drops dramatically. The peaks assigned to the Cγ-H_γ_ correlations (δ_C_/δ_H_ 71.6/3.80 and 4.18 ppm) disappear almost completely.

It is important to note the appearance of a peak at δ_C_/δ_H_ 53.6/5.00 ppm assigned to the CH_3_ or CH groups in the aliphatic region of the sulfated ethanol lignin spectrum. We failed to establish an unambiguous correspondence of this peak to any structural fragment.

The ^1^H–^13^C aromatic region in the HSQC spectrum of the birch ethanol lignin (Figure 9) contains characteristic correlation peaks of the syringyl (S) and guaiacyl (G) units, p-coumarates (pCA), and cinnamyl aldehyde end groups (J). The syringyl units are of several types. In particular, using the assignments made in [28,29,30], we found S units with a substituent in the 4-position (δ_C_/δ_H_ 104.5/6.70), S units with a free hydroxyl group in the 4-position (δ_C_/δ_H_ 106.2/6.51), S units with a carbonyl group in the α-position (δ_C_/δ_H_ 107.1/7.35), and S units with a carboxyl group in the α position (δ_C_/δ_H_ 107.2/7.23).

In addition, there are high-intensity signals at δ_C_/δ_H_ = 129.1/7.73 and 131.9/7.67 ppm corresponding to the CH or CH_3_ groups. Some researchers believe that the signals located at these chemical shifts can be attributed to both C_α,β_−H_α,β_ in stilbenes [31] and C2,6-H_2,6_ in p-benzoates [32].

In the aromatic region of the sulfated lignin spectrum containing the main part of the peaks of the syringyl (S_2,6_eth, S′_2,6_, S″_2,6_) and guaiacyl units (G_2_, G_5_, G_6_), p-coumarates (pCA_3,5_), and cinnamyl aldehyde end groups (J_2,6_) (see Table 6), the signal of the C2,6-H_2,6_ syringyl units with a free hydroxyl group in the 4-position disappears almost completely. This is apparently due to the replacement of this hydroxyl group by the sulfate one. In addition, in this region of the spectrum, a new peak at δ_C_/δ_H_ = 120.8/7.38 and 7.32 ppm appears, which is most likely a signal of the C5-H_5_ guaiacyl (Gs) and C3,5-H_3,5_ p-coumarate (pCAs) structural units with sulfate groups attached to the 4-position. These changes in the chemical shifts of adjacent positions 3 and 5 of the aromatic ring caused by esterification correspond to the expected change of the substituent in phenol [33]. The possible substitution of the sulfate group of the phenolic hydroxyls for the guaiacyl (G) units in the 4-position is evidenced also by the almost complete disappearance of the peak at δ_C_/δ_H_ = 115.7/6.97 ppm characteristic of the C5-H_5_ guaiacyl units (G_5_) with unsubstituted hydroxyl in the 4-position [34].

Based on the data obtained, it can be concluded that sulfation affects the acceptable aliphatic hydroxyl groups of lignin at the γ-positions of β-aryl ethers, α-ethoxylated β-aryl ethers, phenylcoumaran substructures, and cinnamyl alcohol end groups, as well as the unsubstituted hydroxyl groups in the α-position of β-aryl ethers. In addition, free phenolic hydroxyl groups in the 4-position of syringyl and guaiacyl units and p-coumarates can be subjected to sulfation.

The comparison of the 2D NMR spectroscopy data on birch and abies ethanol lignin sulfates [16] revealed higher structural diversity of the sulfated birch ethanol lignin. A significant difference between the HSQC spectra of birch ethanol lignin and abies ethanol lignin in the area of correlations of the aromatic fragments is the presence of several types of syringyl units in the former. However, both samples are built from the phenylpropane structural units linked by simple ether (β-O-4′) and C–C (β–β′, β–5′) bonds, while the sulfate groups are localized mainly in the γ and α positions of the side chains and, probably, in the 4-position of aromatic rings.

**Figure 12:**
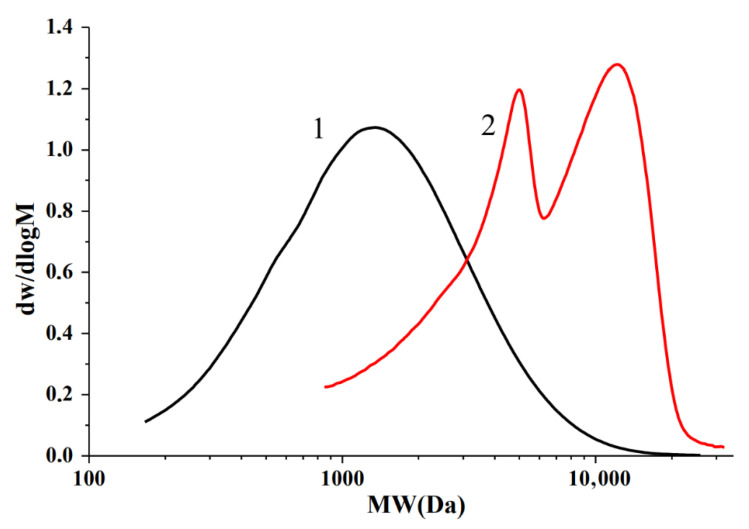
Molecular weight distribution curves for (1) the birch ethanol lignin and (2) sulfated ethanol lignin samples.

Data on the molecular weight distribution of the initial and sulfated birch ethanol lignin were obtained by the GPC method. The molecular weight distribution curves for the initial and sulfated birch ethanol lignin samples are shown in Figure 12. The average molecular weights and polydispersity of the initial and sulfated ethanol lignins are indicated in Table 6.

The birch ethanol lignin obtained in [35] had a low molecular weight (*M*_w_ = 1800 Da) and a monomodal distribution (PD = 2.02), which evidences higher homogeneity of the sample as compared to Alcell birch lignin (*M*_w_ = 3470 and PD = 4.1) [17].

As a result of birch ethanol lignin sulfation, the weight average molecular weight *M*_w_ of the samples increased from ~1800 to ~7600 Da. Such a significant growth is related to an increase in the weight of lignin macromolecules due to the introduction of sulfate groups and the removal of the low molecular weight fraction of the sulfated lignin along with inorganic impurities at the dialysis stage. A feature distinguishing sulfated birch ethanol lignin from abies ethanol lignin sulfated under similar conditions [16] is the bimodal molecular weight distribution (Figure 12). The molecular weight distribution curve of birch ethanol lignin has two pronounced peaks with molecular weights of ~5000 and ~12,000 Da. These peaks can be attributed to the heterogeneity of the initial ethanol lignin molecules, which enter into the sulfation reaction in different ways. The low molecular weight lignin fraction is possibly less sulfated than the high molecular weight fraction, which is reflected in the separation of the peaks in the molecular weight distribution curve. Sulfated birch ethanol lignin has a higher polydispersity and a higher average molecular weight than sulfated abies ethanol lignin (*M*_w_~5300 Da, PD = 1.63) [16].

### 2.4. Thermochemical Properties of the Birch Ethanol Lignin

The thermochemical properties of the birch ethanol lignin were studied using the non-isothermal TG/DTG analysis in an argon medium in the temperature range of 30–900 °C.

**Figure 13:**
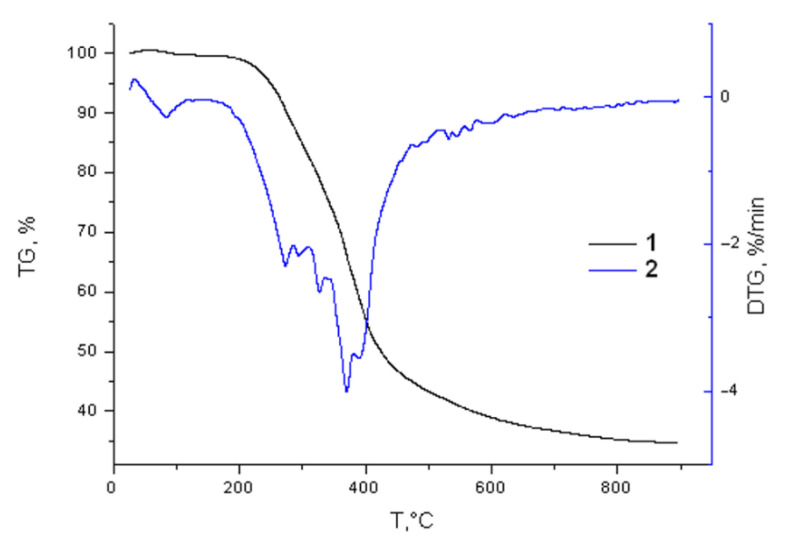
(1) TG and (2) DTG curves for the birch ethanol lignin sample.

The thermal decomposition of the ethanol lignin occurred over a wide temperature range, since its structure contains various functional groups with different thermal stabilities (Figure 13).

The sample weight loss at temperatures of 30–180 °C was found to be less than 1%. This is explained by the loss of moisture and adsorbed gases. The main thermal decomposition of the ethanol lignin started after 200 °C and practically ended at 600 °C. The solid residue yield gradually decreased with an increase in temperature to 700 °C and then remained constant invariable. At a pyrolysis temperature of 900 °C, the solid residue yield was 34.6 wt %, which is somewhat less than in the case of pyrolysis of the abies ethanol lignin under similar conditions (36.2 wt %) [36]. As is known [37], coniferous lignins consist mainly of the guaiacyl structures, while in hardwood lignins, the syringyl structures dominate. The high yield of the carbon residue during the thermal decomposition of abies ethanol lignin is probably due to the tendency of the guaiacyl propane units to condensation reactions [38].

The DTG curve has a broad peak corresponding to the main thermal decomposition of ethanol lignin and an implicit peak. The maximum rate of thermal degradation of the birch ethanol lignin (4.1%/min) was reached at 372 °C. In the temperature range of 350–400 °C, the main lignin structural moieties (guaiacyl and syringyl) underwent cracking with the formation of phenol-type compounds of different molecular weights, the yield of which increased with temperature [39].

At this temperature range, the pyrolysis products represent a complex mixture of organic compounds containing the aromatic, hydroxyl, and alkyl groups and reflecting the composition and structural features of the initial lignin [40]. During the thermal decomposition of the lignin, the competing depolymerization reactions with the formation of lower molecular weight aromatic products and cross-linking reactions of aromatic compounds and their carbonization occurred [40]. In the temperature range of 450–600 °C, the birch ethanol lignin weight loss rate significantly decreased and the thermal decomposition was mainly completed at 600 °C. In this case, some of the aromatic rings in the lignin probably decomposed and condensed into carbon products [41].

**Figure 14:**
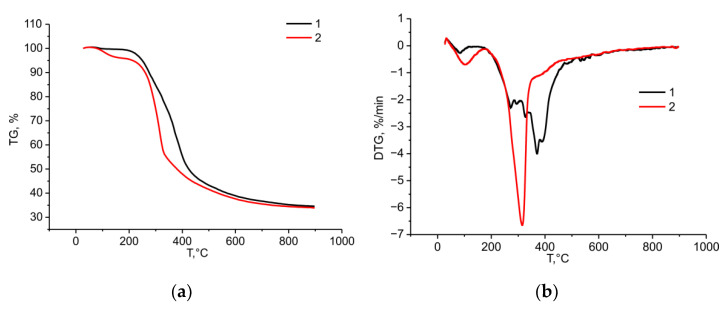
TG (**a**) and DTG (**b**) curves of the birch ethanol lignin (1) and sulfated birch ethanol lignin (2) samples.

The sulfation of the birch ethanol lignin noticeably changed the nature of its thermal transformation (Figure 14).

According to the data presented in Figure 14a, the sulfation of the birch ethanol lignin reduced its thermal stability. At temperature 300 °C, the sulfated ethanol lignin lost 26.4% of its initial weight, while the initial lignin lost only 15.7%. This tendency continued until the completion of the pyrolysis process.

The sulfation of the birch ethanol lignin also changed its thermal transformation profile (Figure 14b). In the temperature range of 100–150 °C, the sulfated ethanol lignin weight loss rate was much higher than in the case of initial ethanol lignin. As was shown in [42], in this temperature range, the aliphatic hydroxyl groups, carbonyl groups, and C–C bonds in the lignin side chains are broken.

In the temperature range of 200–350 °C, an intense narrow peak appeared in the DTG curve of the sulfated birch ethanol lignin, with a maximum weight loss rate of 6.7%/min at 317 °C, which is attributed to the thermal decomposition of sulfate groups [43].

Thus, the TG/DTG study showed that the syringyl structure of hardwood (birch) ethanol lignin was thermally less stable than the guaiacyl structure dominating in softwood (abies) ethanol lignin. Additionally, the introduction of sulfate groups into the structure of birch ethanol lignin reduced its thermal stability.

## 3. Materials and Methods

### 3.1. Materials

The silver birch (*Betula pendula Roth*) wood harvested in the vicinity of Krasnoyarsk city was used as a feedstock for the isolation of ethanol lignin. The contents of the main birch wood components (% of the absolutely dry wood weight) were 47.3 cellulose, 28.5 hemicelluloses, 19.0 lignin, 4.9 extractives, and 0.3 ash.

### 3.2. Ethanol Lignin Isolation

The ethanol lignin was separated from birch wood by extraction with an ethanol–water (60:40) mixture in a Rexo Engineering autoclave reactor (Rexo Engineering Co., Ltd., Seoul, Korea) with a capacity of 3000 mL at a temperature of 185 °C under a working pressure of 0.75 MPa for 3 h and subsequent precipitation with cold water using the technique described in [35].

### 3.3. Ethanol Lignin Sulfation

The obtained ethanol lignin was sulfated with sulfamic acid in 1,4-dioxane in the presence of urea using the procedure described in [16,44].

The sulfation of ethanol lignin was carried out in a three-neck flask (150 mL) equipped with a reflux condenser, a thermometer, and a mechanical stirrer at temperatures 70–100 °C. The ethanol lignin (1.25 g) was added to a mixture of sulfamic acid and urea (mol. ratio 1:1) in 15 mL of 1,4-dioxane. The mixture was intensively stirred for 30–180 min and cooled to room temperature. The solvent was decanted, and the remaining solid product was dissolved in a small amount of water and neutralized with aqueous ammonia to pH 8. To remove the excess reactants, the product was dialyzed against water in a plastic bag of MF-503-46 MFPI brand (USA) with a pore size of 3.5 kDa for 8–10 h. After dialysis, the aqueous solution of sulfated lignin was evaporated with the use of a rotary evaporator to obtain a solid residue—sulfated lignin in the form of an ammonium salt.

The sulfation process of the birch ethanol lignin with sulfamic acid was numerically optimized using the Statgraphics Centurion XVI software, DOE (Design of Experiment) block by the method described in [16].

The estimated sulfated ethanol lignin weight was calculated on the basis of the sulfur content [21] as

(3) $$m_{calc}=\frac{32\times m}{32-0.97\times S}$$

The sulfated product *Yield (%)* was determined as

(4) $$Yield\;(\%)=\frac{m_{actual}}{m_{calc}}\times 100\%$$

where *m_calc_* is the calculated sulfated ethanol lignin weight (g), *m* is the weight of the initial ethanol lignin sample (g), *m_actual_* is the sulfated ethanol lignin weight (g), and *S* is the sulfur content in the sulfated ethanol lignin (%).

### 3.4. Elemental Analysis

The elemental analysis of the sulfated ethanol lignin was carried out using a ThermoQuest FlashEA-1112 analyzer (Milan, Italy).

### 3.5. FTIR Analysis

The FTIR spectra of the initial and sulfated ethanol lignin were recorded using a Shimadzu IRTracer-100 Fourier transform IR spectrophotometer (Tokyo, Japan) in the wavelength range of 400–4000 cm^−1^. The spectral data were processed using the OPUS software (version 5.0). The solid samples for the analysis were tablets in a KBr matrix (2 mg of the sample/1000 mg of KBr).

### 3.6. NMR Analysis

The 2D NMR spectra were recorded at 25 °C in 5-mm ampoules using a Bruker Avance III 600 NMR spectrometer (Billerica, MA, USA) at working frequencies of 600 (^1^H) and 150 MHz (^13^C). Approximately 80 mg of lignin was dissolved in 0.6 mL of deuterated dimethyl sulfoxide, and then the spectra were recorded in the heteronuclear single quantum correlation (HSQC) experiments with editing (HSQCed) using the Bruker standard sequence library. The solvent signal was used as an internal standard (δ_C_ 40.1 and δ_H_ 2.5).

### 3.7. Gel Permeation Chromatography

The number average molecular weight *M*_n_, weight average molecular weight *M*_w_, and polydispersity index of the initial and sulfated ethanol lignin samples were determined by gel permeation chromatography (GPC) using an Agilent 1260 Infinity II Multi-Detector GPC/SEC System with triple detection: refractometer (RI), viscometer (VS), and light scattering (LS). The water-soluble samples were separated on two combined PL Aquagel-OH Mixed-M columns using 0.1 M NaNO_3_ as the mobile phase. The tetrahydrofuran (THF)-soluble samples were separated on a PLgel 10 µm MIXED-E column with the THF mobile phase stabilized with 250 ppm of butylhydroxytoluene. The columns were calibrated using the polyethylene glycol and polystyrene polydisperse standards (Agilent, Santa Clara, CA, USA), respectively. The eluent flow rate was 1 mL/min, and the injected sample volume was 100 µL. Before the analysis, the ethanol lignin and sulfated ethanol lignin samples were dissolved in THF and water (5 mg/mL), respectively; after that, they were filtered through a 0.45-µm Millipore PTFE membrane filter. The data were collected and processed using the Agilent GPC/SEC MDS software.

### 3.8. Thermogravimetric Analysis

The thermogravimetry analysis was carried out using a Netzsch STA 449 F1 Jupiter instrument (Waldkraiburg, Germany). The thermal degradation of the lignin samples was studied in argon in the temperature range from 298 to 1173 K. The samples were heated in the dynamic mode at a heating rate of 10 °C/min in corundum crucibles. The measured data were processed using the Netzsch Proteus Thermal Analysis.5.1.0 software package supplied with the instrument.

### 3.9. Kinetic Calculations

The kinetics of the process of birch ethanol lignin sulfation was studied in the temperature range of 70–100 °C. The apparent initial rates and rate constants of the sulfation reaction were calculated from the change in the sulfur content in the sulfated ethanol lignin. The calculation was carried out according to the first-order equation:

(5) $$V=k\times S=\frac{dS}{dt}$$

where *V* is the rate of the sulfation reaction, wt %/s; *k* is the rate constant of the sulfation reaction, 1/s; *dS* is the change in the sulfur content in birch ethanol lignin sulfate, wt %; and *dt* is the change in time, s. The activation energy was found by the tangent of the slope of the dependence of ln *k* on 11/T.

## 4. Conclusions

As a result of the accomplished study, the main regularities of the process of birch ethanol lignin sulfation with a sulfamic acid–urea mixture in a 1,4-dioxane medium at temperatures of 70–110 °C were established and the sulfating products were characterized using chemical and physical analysis methods.

It was found that similar to the sulfation of abies ethanol lignin under the same conditions [16], the process was complicated by diffusion restrictions due to the increased viscosity of the reaction medium. In the case of excess sulfating agent, the main factors affecting the yield of the sulfated product and sulfur content were the temperature and the duration of the process. Using experimental and computational methods, the optimal conditions for the process of birch ethanol lignin sulfation with a sulfamic acid–urea mixture to provide a high yield of sulfated product (more than 96 wt %) with a sulfur content of 8.1 wt % were established. As in the case of abies ethanol lignin, the sulfation increased the molecular weight of the birch ethanol lignin from 1800 Da to 7600 Da and decreased the polydispersity from 2.02 to 1.81. Moreover, aliphatic hydroxyl groups were more easily sulfated.

Some differences in the sulfation of ethanol lignins of birch and abies were established due to the presence of phenylpropane units of different compositions within these lignins. The sulfation of birch ethanol lignin in which syringyl structures predominate proceeds more completely at moderate temperatures than the abies ethanol lignin with guaiacyl structure. Additionally, in contrast to sulfated abies ethanol lignin, the sulfated birch ethanol lignin had a bimodal and wider molecular weight distribution, as well as less thermal stability.

The sulfated birch ethanol lignin has prospects for use in the production of new sorbents, biocomposites, and nanomaterials, as well as in the development of new anticoagulant and antiviral drugs [12,13,45].

## Figures and Tables

**Table 1:** Effect of the conditions for ethanol lignin sulfation with the sulfamic acid–urea mixture in 1,4-dioxane on the yield of water-soluble sulfated lignin and sulfur content.

No.	L:SC, mol/mol	Temperature, °C	Time, min	Sulfur Content, wt %	Yield, wt %
1	1:3	70	30	2.31 ± 0.02	*
2	1:3	70	45	3.79 ± 0.02	*
3	1:3	70	60	5.22 ± 0.03	94.35 ± 4.04
4	1:3	70	90	5.79 ± 0.03	94.95 ± 3.96
5	1:3	70	120	6.03 ± 0.03	95.56 ± 3.93
6	1:3	70	180	6.31 ± 0.04	95.14 ± 3.88
7	1:3	80	30	2.62 ± 0.02	*
8	1:3	80	45	4.03 ± 0.03	*
9	1:3	80	60	6.08 ± 0.04	93.90 ± 3.91
10	1:3	80	90	6.73 ± 0.03	94.99 ± 3.83
11	1:3	80	120	6.92 ± 0.05	95.53 ± 3.80
12	1:3	80	180	7.09 ± 0.03	96.06 ± 3.77
13	1:3	90	30	3.42 ± 0.02	*
14	1:3	90	45	5.84 ± 0.04	95.61 ± 3.96
15	1:3	90	60	6.93 ± 0.05	96.17 ± 3.80
16	1:3	90	90	7.52 ± 0.05	96.43 ± 3.71
17	1:3	90	120	7.59 ± 0.05	94.82 ± 3.69
18	1:3	90	180	7.92 ± 0.05	94.31 ± 3.65
19	1:3	100	30	4.18 ± 0.03	*
20	1:3	100	45	6.43 ± 0.04	93.50 ± 3.87
21	1:3	100	60	7.44 ± 0.05	93.08 ± 3.72
22	1:3	100	90	7.93 ± 0.05	92.48 ± 3.65
23	1:3	100	120	8.15 ± 0.05	91.74 ± 3.62
24	1:3	100	180	8.14 ± 0.05	91.14 ± 4.07
25	1:3	110	30	5.02 ± 0.03	92.31 ± 3.93
26	1:3	110	45	6.91 ± 0.04	91.74 ± 3.80
27	1:3	110	60	7.90 ± 0.05	90.66 ± 3.65
28	1:3	110	90	8.11 ± 0.05	89.93 ± 3.62
29	1:3	110	120	8.10 ± 0.05	88.73 ± 3.62
30	1:3	110	180	8.13 ± 0.05	85.71 ± 3.62

*—Sulfated lignin with a sulfur content of ≤ 4.20 wt % is water-insoluble.

**Table 2:** Apparent rate constants and activation energies of the process of birch ethanol lignin sulfation with the sulfamic acid–urea mixture.

Temperature	Apparent Initial Rate Constant, K × 10^−4^ (s^−1^)	Activation Energy, kJ/mol
70	1.41	10.7
80	1.80	
90	2.05	
100	2.78	

**Table 3:** Designations of independent variables (factors) and output parameters (experimental results).

Factors and Parameters	Designations in the Equations
Temperature, °C	*X*_1_
Time, h	*X*_2_
Sulfur content, %	*Y*_1_
Product yield, %	*Y*_2_

**Table 4:** Results of the variance analysis.

Variance Source	Output Parameters			
	Sulfur Content *Y*_1_		Yield *Y*_2_	
	Statistical Characteristics			
	Variance Relation *F*	Significance Level *P*	Variance Relation *F*	Significance Level *P*
*X*_1_	78.74	0.0000	200.98	0.0000
*X*_2_	24.87	0.0001	10.88	0.0042
*X*_1_^2^	9.84	0.0060	59.66	0.0000
*X*_1_ *X*_2_	0.00	0.9757	36.11	0.0000
*X*_2_^2^	21.54	0.0002	0.26	0.6184
*R* ^2^ *_adj_*	86.1		92.8	

**Table 5:** Assignment of the ^1^H–^13^C peaks in the HSQC spectra of the initial and sulfated birch ethanol lignins.

Designation	δ_C_/δ_H_, ppm (Initial Lignin)	δ_C_/δ_H_, ppm (Sulfated Lignin)	Assignment
OMe	56.3/3.74	56.3/3.76	C-H in the methoxy groups (OMe)
A_γ_ and A′_γ_	60.0/3.42−3.74	-	C_γ_-H_γ_ in the β-aryl ether (β-O-4′) substructures (A) and α-ethoxylated (C_α_OEt) β-aryl ether (β-O-4′) substructures (A′)
A_γ_s and A′_γ_s	-	64.6/3.51 and 3.83	C_γ_-H_γ_ in the γ-sulfated (γ-OSO_3_NH_4_) β-aryl ether (β-O-4′) substructures (As) and γ-sulfated (γ-OSO_3_NH_4_) α-ethoxylated (C_α_OEt) β-aryl ether (β-O-4′) substructures (A′s)
A_β(G)_ and A′_β(G)_	84.1/4.30 and 83.4/4.39	-	Cβ-H_β_ in the β-aryl ether (β-O-4′) substructures bonded to the G units (A) and α-ethoxylated (C_α_OEt) β-aryl ether (β-O-4′) substructures bonded to the G units (A′)
A_β(S)_ and A′_β(S)_	86.5/4.12 and 84.8/4.27	-	Cβ-H_β_ in the β-aryl ether (β-O-4′) substructures bonded to the S units (A) and α-ethoxylated (C_α_OEt) β-aryl ether (β-O-4′) substructures boded to the S units (A′)
A_β(G)_s and A′_β(G)_s	-	80.7/4.50 and 80.0/4.64	Cβ-H_β_ in the γ-sulfated (γ-OSO_3_NH_4_) β-aryl ether (β-O-4′) substructures bonded to the G units (As) and γ-sulfated (γ-OSO_3_NH_4_) α-ethoxylated (C_α_OEt) β-aryl ether (β-O-4′) substructures bonded to the G units (A′s)
A_β(S)_s and A′_β(S)_s	-	82.1/4.35 and 82.1/4.50	Cβ-H_β_ in the γ-sulfated (γ-OSO_3_NH_4_) β-aryl ether (β-O-4′) substructures boded to the S units (As) and γ-sulfated (γ-OSO_3_NH_4_) α-ethoxylated (C_α_OEt) β-aryl ether (β-O-4′) substructures boded to the S units (A′s)
A_α_	72.5/4.88	-	C_α_-H_α_ in the β-aryl ether (β-O-4′) substructures (A)
A′_α_OEt	64.4/3.33	64.7/3.36	C-H of the methylene groups in the α-ethoxylated (C_α_OEt) β-aryl ether (β-O-4′) substructures (A′)
A′_α_	81.2/4.56	81.1/4.53	C_α_-H_α_ in the α-ethoxylated (C_α_OEt) β-aryl ether (β-O-4′) substructures (A′)
B_β_	54.1/3.06	55.1/3.06	Cβ-H_β_ in the pinoresinol (β–β′) substructures (B)
B_γ_	71.6/3.80 and 4.18	-	C_γ_-H_γ_ in the pinoresinol (β–β′) substructures (B)
B_α_	85.6/4.68	85.9/4.68	C_α_-H_α_ in the pinoresinol (β–β′) substructures (B)
C_β_	53.7/3.48	-	Cβ-H_β_ in the phenylcoumaran (β–5′) moieties (C)
C_β_s	-	51.1/3.65	Cβ-H_β_ in the γ-sulfated (γ-OSO_3_NH_4_) phenyl coumaran (β–5′) moieties (Cs)
C_γ_	63.4/3.74	-	C_γ_-H_γ_ in the phenyl coumaran (β–5′) substructures (C)
C_γ_s	-	67.5/3.94	Cγ-H_γ_ in the γ-sulfated (γ-OSO_3_NH_4_) phenylcoumaran (β–5′) substructures (Cs)
C_α_	87.6/5.45	87.5/5.55	Cα-H_α_ in the phenylcoumaran (β–5′) substructures (C_α_)
I_γ_	60.4/4.03	59.9/4.03	Cγ-H_γ_ in the cinnamyl alcohol end groups (I)
I_γ_s	-	64.6/4.15	Cγ-H_γ_ in the γ-sulfated (γ-OSO_3_NH_4_) cinnamyl alcohol end groups (Is)
J_2,6(S)_	107.0/7.08	106.4/7.00	C2,6-H_2,6_ in the cinnamyl aldehyde end groups (J)
S_2,6_eth	104.5/6.70	104.8/6.62	C2,6-H_2,6_ in the 4-etherified syringyl units (Seth)
S_2,6_	106.2/6.51	-	C2,6-H_2,6_ in the 4-non-etherified syringyl units (S)
S′_2,6_	107.1/7.35	106.7/7.30	C2,6-H_2,6_ in the oxidized (C_α_=O) syringyl units (S′)
S″_2,6_	107.2/7.23	106.5/7.20	C2,6-H_2,6_ in the oxidized (C_α_OOH) syringyl units (S″)
pCA_3,5_	115.8/6.79	115.5/6.79	C3,5-H_3,5_ in the p-coumarates (pCA)
pCA_3,5_s	-	120.8/7.32	C3,5-H_3,5_ in the 4-sulfated (4-OSO_3_NH_4_) p-coumarates (pCAs)
G_2_	110.6/6.94	111.9/7.00	C2-H_2_ in the 4-etherified guaiacyl units (Geth)
G_5_	115.7/6.97	-	C5-H_5_ in the 4-non-etherified guaiacyl units (G)
G_5_s	-	120.8/7.38	C5-H_5_ in the 4-sulfated (4-OSO_3_NH_4_) guaiacyl units (Gs)
G_5_eth	115.7/6.76	115.4/6.76	C5-H_5_ in the 4-etherified guaiacyl units (Geth)
G_6_	119.4/6.79	119.5/6.76	C6-H_6_ in the 4-etherified guaiacyl units (Geth)

**Table 6:** Average molecular weights *M*_n_ and *M*_w_ and polydispersity of the initial and sulfated birch ethanol lignin samples.

Sample	*M*_n_ (Da)	*M*_w_ (Da)	PD
Birch ethanol lignin	902	1828	2.02
Sulfated birch ethanol lignin	4199	7599	1.81

## Data Availability

All data generated during this study are included in the article.

## Sample Availability

Samples of the compounds are available from the authors.
